# Non-standard management of breast cancer increases with age in the UK: a population based cohort of women ⩾65 years

**DOI:** 10.1038/sj.bjc.6603709

**Published:** 2007-03-27

**Authors:** K Lavelle, C Todd, A Moran, A Howell, N Bundred, M Campbell

**Affiliations:** 1 School of Nursing, Midwifery and Social Work, The University of Manchester, Manchester, UK; 2 School of Nursing, Midwifery and Social Work, The University of Manchester, Manchester, UK; 3 North West Cancer Intelligence Service (NWCIS), Central Manchester PCT, Manchester, UK; 4 CRUK Department of Medical Oncology, Christie Hospital NHS Trust, Manchester, UK; 5 Withington Hospital, South Manchester University Hospitals NHS Trust, Manchester, M20 2LR, UK; 6 School of Nursing, Midwifery and Social Work, The University of Manchester, Manchester, UK

**Keywords:** breast cancer, elderly, treatment, diagnosis, tumour characteristics

## Abstract

Evidence suggests that compared to younger women, older women are less likely to receive standard management for breast cancer. Whether this disparity persists once differences in tumour characteristics have been adjusted for has not been investigated in the UK. A retrospective cohort study involving case note review was undertaken, based on the North Western Cancer Registry database of women aged ⩾65 years, resident in Greater Manchester with invasive breast cancer registered over a 1-year period (*n*=480). Adjusting for tumour characteristics associated with age by logistic regression analyses, older women were less likely to receive standard management than younger women for all indicators investigated. Compared to women aged 65–69 years, women aged ⩾80 years with operable (stage 1–3a) breast cancer have increased odds of not receiving triple assessment (OR=5.5, 95% confidence interval (CI): 2.1–14.5), not receiving primary surgery (OR=43.0, 95% CI: 9.7–191.3), not undergoing axillary node surgery (OR=27.6, 95% CI: 5.6–135.9) and not undergoing tests for steroid receptors (OR=3.0, 95% CI: 1.7–5.5). Women aged 75–79 years have increased odds of not receiving radiotherapy following breast-conserving surgery compared to women aged 65–69 years (OR=11.0, 95% CI: 2.0–61.6). These results demonstrate that older women in the UK are less likely to receive standard management for breast cancer, compared to younger women and this disparity cannot be explained by differences in tumour characteristics.

The highest incidence of breast cancer in England occurs in women aged 70 years and older (ONS, 2002). Older women also experience the worst survival. Women aged 70–79 years have a 76% 5-year relative survival compared to 80% for all ages. For patients aged ⩾80 years, survival drops considerably to 61%, beyond what might be expected owing to an increase in age (ONS, 2005). It is important, therefore, to investigate patterns of management for possible explanations for this.

Compared to younger women, older women with breast cancer are less likely to be diagnosed via needle biopsy and triple assessment, less likely to undergo primary and axillary node surgery, less likely to receive radiotherapy following breast-conserving surgery, and less likely to receive chemotherapy (Busch *et al*, 1996; Hérbert-Croteau *et al*, 1999; Mandelblatt *et al*, 2000; Bouchardy *et al*, 2003; Gennari *et al*, 2004; Wyld *et al*, 2004; Giordano *et al*, 2005). Such management of older women is likely to lead to poor local control, recurrence of the disease and excess mortality (COIN, 1999; BASO, 2005; SIGN, 2005).

The extent to which tumour characteristics can explain difference in management is of primary importance. Older women could legitimately receive different treatment to younger women owing to, for example, larger tumour size or differences in receptor status (COIN, 1999; SIGN, 2005). Although tumour characteristics provide a basis for treatment decisions in published guidelines, how tumour characteristics vary with age remains uncertain (Fisher *et al*, 1997; Diab *et al*, 2000). There is evidence that time between onset of symptoms of breast cancer and first hospital visit is greater for older patients and that variation in some tumour characteristics by age, such as stage and tumour size, is related to that delay (Ramirez *et al*, 1999; Richards *et al*, 1999). However, regardless of why tumour characteristics differ between age groups, once older women present with breast cancer they are less likely to receive standard management. It is important, therefore, to establish the extent to which tumour characteristics account for this in epidemiological, multivariate studies, which adjust for the possible confounding effects of variation in tumour characteristics.

Studies undertaken so far in the UK have been limited to univariate investigation of the relationship between age and treatment. Two studies based in England found that older women with early stage breast cancer were less likely to receive radiotherapy following breast-conserving surgery, axillary node surgery and chemotherapy and more likely to receive hormone therapy as their sole form of treatment (Golledge *et al*, 2000; Wyld *et al*, 2004). An audit of symptomatically presenting breast cancer patients also identified considerable difference in several aspects of management between younger and older women (Monypenny, 2004). However, none of these UK studies adjusts for the possible confounding effects of differences in tumour characteristics on treatment choice in multivariate models. Therefore the extent to which tumour characteristics account for under treatment of older women with breast cancer in the UK, has not been established.

The evidence from such studies conducted in North American (Hérbert-Croteau *et al*, 1999; Mandelblatt *et al*, 2000; Du and Goodwin, 2001; Yancik *et al*, 2001; DeMichele *et al*, 2003; Giordano *et al*, 2005) and to a lesser extent mainland Europe (Bouchardy *et al*, 2003; Nagel *et al*, 2003; Gennari *et al*, 2004) suggests that age predicts non-standard management when tumour characteristics are accounted for. However, no similar studies have been undertaken in the UK and these results may not be representative of practice in the UK, especially in light of the comparatively lower survival rate of women aged ⩾65 years compared to the rest of Europe (Vercelli *et al*, 2000) and the United States (ONS, 2002; Ries *et al*, 2003) and differences in health care services between countries.

In addition, previous studies have not investigated variation in receipt of steroid receptor testing by age group amongst older women with breast cancer. As the results of this test determines suitability for hormone therapy and reliance on this method of treatment alone rises with increasing age group (Wyld *et al*, 2004), it is important to establish if testing varies with age.

This study, therefore, investigates whether age predicts a range of indicators of standard management, including receptor testing, amongst a population of women, registered with invasive breast cancer, aged ⩾65years and resident in Greater Manchester (UK) accounting for tumour characteristics using logistic regression analysis.

## MATERIALS AND METHODS

Minimal standards of acceptable management for non-metastatic breast cancer were developed based on published guidelines (BASO, 1998; SIGN, 1998; COIN, 1999; NWBG, 1999) in consultation with two consultant breast surgeons, a medical and a clinical oncologist and an epidemiologist. ‘Operable breast cancer’ is defined as presenting with UICC stage 1–3a assessed on the basis of diagnostic procedures undertaken before therapeutic surgery (SIGN, 1998; NWBG, 1999) (see Box 1).

Pretreatment assessments of tumour characteristics, tumour size and stage were recorded based on clinical and imaging assessments (cTNM). Overall assessments of these tumour characteristics were based on postsurgery pathological assessment (pTNM), if undertaken and pretreatment assessment if not (UICC, 1997).

To test the null hypothesis that older women are as likely to receive these standards of management, whilst accounting for tumour characteristics, we undertook an observational retrospective cohort study. Our sample included all women aged ⩾65 years old, resident in Greater Manchester, with Cancer Registry anniversary dates for invasive breast cancer during 1999 (see Box 2).

Case note reviews were undertaken in order to check and supplement Cancer Registry information on management, tumour variables and age. All cases were followed to 31 December 2001. A proforma to collect information from case notes was developed and checked for inter-observer and test–retest reliability using Cohen's *κ* statistic in 3% of cases reviewed, chosen at random. In addition, quality checks of approximately 10% of the database entries against the original proformas were undertaken.

Univariate analysis of categorical variables used the Pearson's *χ*^2^ test and the *χ*^2^ test for trend. All tests are two-tailed with *α*=0.05 unless otherwise specified.

Significant indicators of standard management associated with age in univariate analysis are used as independent variables in the subsequent logistic regression (non-stepwise). All logistic regression models meet the recommendation of at least 10 cases per variable (Norman and Streiner, 2000). In practice, there were no problems with convergence of the maximum likelihood estimates (Tabachnick and Fidell, 2001). To meet this recommendation the following strategy was used to select explanatory variables. All logistic regression models include the variables of age group, hospital type (university *vs* district) and deprivation (Townsend index quintiles 1–2 *vs* 3–5). Tumour characteristics selected for entry into the logistic regression models had to be significantly associated with age in the univariate analysis, known to the clinician at the time the management decision was made, have sufficient number of cases with data and not be highly associated with each other.

Data were analysed using SPSS 11.5 for Windows; 95% confidence intervals for percentages were estimated using the CIA (confidence interval analysis) Programme version 1.2 (Gardner *et al*, 1992).

## RESULTS

### Selection bias

Data from the Cancer Registry revealed that the age group of patients whose notes were not reviewed (*n*=136) did not differ significantly from that of the study sample (*n*=480) (Trend *χ*^2^=3.04; df=1; *P*=0.081). However, cases not reviewed were less likely to have surgery (Pearson's *χ*^2^=26.54; df=1; *P*<0.001), indicating that the study sample over represents those receiving surgery. Nonetheless, older women are significantly less likely to receive surgery for both cases not reviewed (Trend *χ*^2^=17.59; df=1; *P*<0.001) and the study sample (Trend *χ*^2^=97.54; df=1; *P*<0.001), demonstrating that the pattern of decreasing surgery with increasing age would still have been found if the complete study sample of all registered patients had been achieved.

### Reliability

Inter- and intra-rater agreement levels of the proformas all satisfied *κ* >0.6, indicating substantial to perfect agreement levels (Landis and Koch, 1977).

### Overall proportions receiving non-standard management

Substantial proportions of all patients in the study failed to receive standard management with the proportions not receiving standard management ranging between 19% for triple assessment to 41% for steroid receptor testing (Table 1).

Of the 169 patients who did not undergo steroid receptor testing, 75% (127) were still treated with tamoxifen, 44% (74) did not undergo surgery and for 32% (54) tamoxifen was their sole form of treatment (i.e. no surgery, radiotherapy or chemotherapy).

Of the 243 patients who did undergo steroid receptor testing, 15% (36) were found to be negative for both oestrogen (ER) and progesterone (PR) receptors. Thirty-five of these patients were still treated with hormone therapy. However, all 36 patients also received some other form of treatment with 32 undergoing surgery.

### Univariate analysis

**Figure 1:**
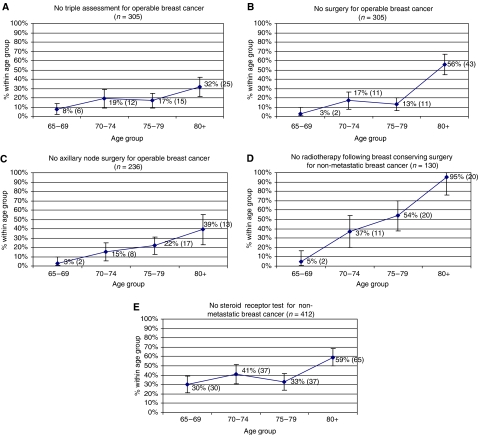
Percentage (*n*) of breast cancer patients not receiving standard management by age group. (**A**) No triple assessment for operable breast cancer (*n*=305). (**B**) No surgery for operable breast cancer (*n*=305). (**C**) No axillary node surgery for operable breast cancer (*n*=236). (**D**) No radiotherapy following breast-conserving surgery for non-metastatic breast cancer (*n*=130). (**E**) No steroid receptor test for non-metastatic breast cancer (*n*=419).

The results of the univariate analysis are presented in Figure 1. The proportions of women not receiving standard management increased with age for all indicators of standard management. The difference in standard management between age groups was significant for all indicators of standard management, as were the tests of trend (*P*<0.001). Even when presenting with operable breast cancer, older women are less likely to be diagnosed by triple assessment, less likely to undergo any surgery within 3 months of diagnosis, less likely to have axillary node dissection as part of this surgery and less likely to have any treatment other than hormone therapy. In addition, older women are less likely to undergo a steroid receptor test within 4 months of diagnosis and less likely to have radiotherapy following breast-conserving surgery.

### Multivariate analysis

The results of the multivariate analysis are given in Table 2. Patients aged ⩾80 years with operable breast cancer have more than five times the odds of not receiving triple assessment for operable breast cancer, compared to the reference group of 65–69 year olds, controlling for pretreatment assessment of tumour size (mm) (OR=5.5 95% CI: 2.1–14.5). Patients aged 70–74 and 75–79 have 7.9 (95% CI: 1.4–44.4) and 11.0 (95% CI: 2.0–61.6) times the odds, respectively, of not undergoing radiotherapy following breast-conserving surgery as compared to 65–69 year olds, controlling for tumour grade (I, II or III) and overall tumour stage. Patients aged ⩾80 years were found to have 3.0 (95% CI: 1.7–5.5) times the odds of not receiving a steroid receptor test within 4 months of diagnosis compared to 65–69 year olds, controlling for overall tumour stage.

Patients aged 70–74 have 6.7 (95% CI: 1.4–32.6) and patients aged ⩾80 years have 43.0 (95% CI: 9.7–191.3) times the odds of not receiving surgery for operable breast cancer compared to 65–69 year olds. All age groups ⩾70 years have significantly increased odds of not receiving axillary node surgery. These odds increase with age up to patients aged ⩾80 years who have 27.6 (95% CI: 5.6–135.9) times the odds of not having axillary node surgery compared with 65–69 year olds. These logistic regressions account for pretreatment stage.

Tumour characteristics were found to be predictive of not receiving standard management. Patients with pretreatment stage 2 tumours are less likely to receive surgery compared to patients with stage 1 tumours (OR=2.8 95% CI: 1.3–6.0) and the odds of not receiving triple assessment increase by 1.02 per mm increase in pretreatment tumour size (95% CI: 1.00–1.04).

Hospital type was predictive of not undergoing triple assessment and not receiving surgery for operable cancers. District hospitals performed less well than university hospitals (triple assessment OR=4.5 95% CI: 2.0–9.9; surgery OR=2.2 95% CI: 1.0–4.5).

## DISCUSSION

We present evidence that, even when differences in the nature of the disease (as measured by tumour characteristics associated with age) are accounted for, older women in Greater Manchester are less likely to receive standard management for breast cancer compared to younger women. Furthermore, there were age trends in our data with the oldest women fairing least well. Compared with her 65–69-year-old counterpart, the odds of a women aged 80 or older not receiving triple assessment for operable breast cancer are five and a half times higher, and the odds of her not receiving surgery are more than 40 times higher, controlling for social deprivation, hospital type as well as size and grade of tumour respectively.

For triple assessment, primary surgery and steroid receptor testing, there appears to be a threshold effect, with women aged < 80 years being treated similarly, but ⩾80 years not receiving timely diagnostic testing and being treated by hormone therapy alone. For axillary surgery and radiotherapy the pattern is more linear, as age increases the odds of standard treatment decreases.

The results of this study are in broad agreement with previous studies from North America (Hérbert-Croteau *et al*, 1999; Mandelblatt *et al*, 2000; Giordano *et al*, 2005) and mainland Europe (Bouchardy *et al*, 2003; Nagel *et al*, 2003; Gennari *et al*, 2004) as they demonstrate an increase in non-standard management once tumour characteristics are accounted for. The two UK studies, described earlier, demonstrated similar age related gradients in non-standard management. However, the possible confounding effects of variation in tumour characteristics on management were not adjusted for in multivariate analysis. This study builds on previous work by demonstrating that even when tumour characteristics are accounted for increasing age predicts non-standard management of postmenopausal women in a UK based population.

In addition, unlike previous studies, patterns of steroid receptor testing by age group were investigated. The percentage not receiving a receptor test varied the least between age groups compared to the other indicators of standard management with 30% of the youngest women (65–69 years) compared to 41% of all age groups in this study not undergoing receptor testing. The overall percentage not receiving receptor testing seems high, as steroid receptor testing was recommended in guidelines in use in clinics at the time of the study (BASO, 1998; SIGN, 1998; COIN, 1999; NWBG, 1999), and suggests that further studies including women <65years old are needed to establish what proportions of younger women undergo receptor testing. Audit data for screen detected breast cancers in 2000/1 do however suggest that, among the screened population (predominantly aged 50–64 year olds), only 12% of women with invasive breast cancer in the UK and 6% in Greater Manchester did not undergo steroid testing for ER receptors (Lawrence and George, 2001).

Nevertheless, failure to investigate receptor status among patients in this study resulted in treatment decisions being made without fundamental information. As 75% of patients not receiving a steroid receptor test were still treated with tamoxifen they were prescribed a treatment without this evidence that it would work and for 32% of these patients it was the only therapy they received. Moreover, all but one of the 36 patients, who were found to be negative for both ER and PR receptors, were still treated inappropriately with tamoxifen. Given the toxicity of tamoxifen in this age group regarding increased risks of thromboembolic events and endometrial cancer (Fisher *et al*, 2005), this practice is potentially dangerous. However, all 36 patients received some other form of treatment and for 32 patients this was surgery. This suggests that although receptor testing does not deter inappropriate hormonal treatments it does discourage reliance on treatment by hormone therapy alone.

Limitations of this study include potential selection bias, restriction of the sample to one geographical region and patient preferences and health status not being taken into account.

Selection bias, owing to the proportion of cases not reviewed, may limit the generalisability of the results of this study. However, a high proportion (593 of the 729 registered cases) were reviewed (81%). Moreover, a selection bias analysis indicates that the age range in the sample is similar to the population and that, although the sample overestimates the proportion received surgery, a similar pattern of decreasing standard management with age would have been found, if the complete sample was achieved.

This study included only one region of England. The extent to which we can generalise these results nationally may therefore be limited especially as geographical variations in survival rates, as well as access to diagnostic and treatment services for cancer have been identified in England (NAO, 2004). However, as the variation in survival by Strategic Health Authority is less for breast cancer than other major cancers, these results may be more applicable nationally than regional studies of other cancers.

Patient preferences were not accounted for in this study and may explain some of the difference in treatment between younger and older women. Yellen *et al* (1994) found that older patients were as likely as their younger counterparts to agree to aggressive therapy such as chemotherapy following surgery. Conversely, other researchers have reported that older women with breast cancer are more likely than younger women not to want further therapies after surgery (Newcomb and Carbone, 1993; Mandelblatt *et al*, 2000). Mandelblatt *et al* (2000), however, still found that age predicted non-receipt of radiotherapy following breast-conserving surgery, even when these patient preferences were accounted for. Clearly, more UK-based research is needed to investigate the influence of patient preference on the disparity in management identified in this study. However, although patient preferences may play a part in treatment decisions for surgery and radiotherapy, patient choice is less likely to be an influence in diagnostic services such as triple assessment and the decision to send a specimen for receptor testing rests with the clinician. Without knowledge of steroid receptor status it is not possible for the clinician or the patient to make an informed decision on choice of treatment.

The difference in management between older and younger women with breast cancer may also be related to the patients’ fitness for treatment. This has been cited in the guidelines as a legitimate reason for non-standard management of older women (SIGN, 1998; NWBG, 1999). Several studies provide evidence that this disparity in treatment still occurs when comorbidity is accounted for (Hérbert-Croteau *et al*, 1999; Du and Goodwin, 2001; Yancik *et al*, 2001; DeMichele *et al*, 2003; Nagel *et al*, 2003; Gennari *et al*, 2004; Giordano *et al*, 2005). However, few control for any further measures of general health (Silliman *et al*, 1997; Mandelblatt *et al*, 2000; Hurria *et al*, 2003). Moreover, no similar studies from the UK have been reported to date. Further studies, accounting for wider measures of health as well as comorbidity and tumour characteristics, based on a UK sample of breast cancer patients are needed.

Despite these limitations, our results reinforce and add weight to the body of literature identifying the disparity in the treatment of older and younger women with breast cancer. The strengths of this study include the use of a registry-based population of all breast cancer patients, measurement of a range of indicators of standard management and incorporation of the possible confounding effects of systematically selected tumour characteristics as well as social deprivation and hospital type in a logistic regression analysis. To the best of our knowledge this is the only UK-based study to do this. We found that older women do not receive the same management as younger women and that this is owing to their age rather than differences in tumour status.

Despite this, in a survey of UK breast surgeons, 75% reported that they would treat older breast cancer patients in a similar way to younger breast cancer patients and 98% responded that the cutoff point for primary surgery was ‘not age related’ (Audisio *et al*, 2004). Clearly there is an apparent difference in clinicians’ perceptions of how older breast cancer patients are treated and actual practice.

Standard management of breast cancer was infrequent in older women in Greater Manchester. The lack of diagnostic and steroid receptor testing resulted in older cancer patients having no effective treatment with 41% not undergoing a steroid receptor test, 32% of whom received tamoxifen as their sole form of treatment. Mortality of elderly breast cancer patients is unlikely to improve where this pattern of management persists.

## Figures and Tables

**Box 1:** Minimal standards of management for non-metastatic invasive breast cancer

*Triple assessment*
All operable^*^ invasive breast carcinomas are diagnosed via clinical assessment, imaging and fine needle aspiration or core biopsy. All three tests need to take place within 1 month.

*Surgery*
Surgery should be undertaken, within 3 months of initial diagnosis, for all cases of operable^*^ breast cancer.

*Axillary node surgery*
Operable^*^ cases undergoing surgery should have some form of axillary node dissection.

*Radiotherapy following breast conserving surgery*
All patient undergoing breast conserving surgery, and not going on to have a mastectomy, should receive radiotherapy within 6 months of their last breast conserving surgery.

*Receptor testing:*
All patients should have a steroid receptor test, within 4 months of initial diagnosis, to assess their suitability for hormone therapy.

^*^‘Operable breast cancer’ =UICC stage 1–3a at presentation on basis of diagnostic procedures before therapeutic surgery undertaken (cTNM)(UICC, 1997; SIGN, 1998)

**Box 2:** Selection of sample

Study sample (*n*=729)	All women aged ⩾65 years, resident in Greater Manchester, recorded on Cancer Registry with an anniversary date for invasive breast cancer in 1999.
Exclusions (*n*=249)	Case notes not available for review (*n*=136); pilot study – randomly selected (*n*=33); metastatic breast cancer diagnosed at presentation (*n*=51); Diagnosis and/or hormone therapy pre 7 July 1998 (*n*=21); Anniversary date pre 1 January 1999 (*n*=2); Ductal carcinoma *in situ* (*n*=4); Moved into/away from Greater Manchester during treatment (*n*=2).
Final sample	*n*=480

**Table 1:** Proportion of all patients in the study not receiving standard management for each indicator

**Indicator of standard management (baseline number)**	**% (*n*) not receiving standard management**	**95% confidence interval**
Triple assessment (*n*=305)	19.0 (58)	14.6–23.4
Surgery (*n*=305)	22.0 (67)	17.3–26.6
Axillary node surgery (*n*=236)	16.9 (40)	12.2–21.7
Radiotherapy following breast conserving surgery (*n*=130)	40.8 (53)	32.3–49.2
Steroid testing (*n*=412)	41.0 (169)	36.3–45.8

**Table 2:** Odds ratios (95%CI) of not receiving standard of management from logistic regression models accounting for tumour characteristics

				**95% CI for odds ratio**	
**Variables**		***P*-value**	**Odds ratio**	**Lower**	**Upper**
*Standard of management: Triple Assessment (*n*=305) Not receiving triple assessment (vs* *receiving triple assessment) for operable breast cancer*					
Pretreatment tumour size (mm)		0.032	1.02	1.00	1.04
Type of hospital	University		1.00		
	District	<0.001	4.46	2.00	9.94
Townsend index (quintiles)	1–2		1.00		
	3–5	0.971	1.01	0.53	1.92
Age group (years)	Overall	0.002			
	65–69 (reference)		1.00		
	70–74	0.092	2.52	0.86	7.40
	75–79	0.187	2.01	0.71	5.64
	80+	0.001	5.49	2.08	14.45
					
*Standard of management: Surgery (*n*=305) Not receiving surgery (vs* *receiving surgery) for operable breast cancer*					
Pretreatment stage (UICC)	Overall	0.027			
	1		1.00		
	2	0.008	2.81	1.31	6.04
	3a	0.195	3.20	0.55	18.63
Type of hospital	University		1.00		
	District	0.042	2.15	1.03	4.48
Townsend Index (quintiles)	1–2		1.00		
	3–5	0.864	0.94	0.46	1.93
Age group (years)	Overall	<0.001			
	65–69 (reference)		1.00		
	70–74	0.018	6.73	1.39	32.58
	75–79	0.060	4.46	0.94	21.19
	80+	<0.001	43.03	9.68	191.25
					
*Standard of management: Axillary node surgery* (n=*236) Not receiving axillary node surgery (vs* *receiving axillary node surgery) for operable breast cancer*					
Pretreatment stage (UICC)	Overall	0.877			
	1		1.00		
	2	0.852	0.93	0.43	2.01
	3a	0.613	0.54	0.05	5.91
Type of hospital	University		1.00		
	District	0.050	2.27	1.00	5.17
Townsend index (quintiles)	1–2		1.00		
	3–5	0.431	0.72	0.31	1.64
Age group (years)	Overall	<0.001			
	65–69(reference)		1.00		
	70–74	0.012	7.85	1.56	39.36
	75–79	0.002	11.76	2.54	54.50
	80+	<0.001	27.59	5.60	135.87
					
*Standard of management: Radiotherapy (*n*=130) Not receiving radiotherapy within 6 months of breast conserving surgery (vs* *receiving radiotherapy)*					
Overall tumour stage	1		1.00		
	2–3	0.444	0.65	0.21	1.98
Tumour grade	Overall	0.306			
	I		1.00		
	II	0.148	0.37	0.10	1.42
	III	0.751	0.79	0.18	3.49
Type of hospital	University		1.00		
	District	0.630	1.32	0.42	4.13
Townsend index (quintiles)	1–2		1.00		
	3–5	0.218	2.48	0.58	10.54
Age group (years)	Overall	<0.001			
	65–69 (reference)		1.00		
	70–74	0.019	7.89	1.40	44.43
	75–79	0.007	10.97	1.95	61.59
	80+	<0.001	406.48	26.07	6337.48
*Standard of management: Receptor testing (*n*=412) Not receiving a steroid receptor test within 4 months of diagnosis (vs* *receiving a receptor test within 4 months)*					
Overall tumour stage	1		1.00		
	2–3	0.709	1.09	0.69	1.71
Type of hospital	University		1.00		
	District	0.076	1.52	0.96	2.42
Townsend index (quintiles)	1–2		1.00		
	3–5	0.150	0.71	0.45	1.13
Age group (years)	Overall	0.001			
	65–69(reference)		1.00		
	70–74	0.167	1.54	0.83	2.85
	75–79	0.906	1.04	0.56	1.90
	80+	<0.001	3.02	1.66	5.52
